# Ischemic mitral regurgitation: To repair or replace? A single center experience

**DOI:** 10.1371/journal.pone.0307449

**Published:** 2024-10-24

**Authors:** Joseph C. Sweeney, Amal Alotaibi, Gene D. Porter, Divya Avula, Jaimin R. Trivedi, Mark S. Slaughter, Brian L. Ganzel, Siddharth V. Pahwa

**Affiliations:** 1 Department of Cardiothoracic Surgery, University of Louisville, Louisville, Kentucky, United States of America; 2 School of Medicine, University of Louisville, Louisville, Kentucky, United States of America; 3 Department of Surgery, University of Kentucky, Lexington, Kentucky, United States of America; BSMMU: Bangabandhu Sheikh Mujib Medical University, BANGLADESH

## Abstract

**Objective:**

Recent reports on ischemic mitral valve (MV) regurgitation surgical strategies have suggested better hemodynamic performance with MV replacement (MVR) than MV repair (MVr) with no survival difference at 2 years. We evaluated the difference between MVR and MVr outcomes in patients with ischemic MR, including hemodynamic MV performance at 1 and 2 years postoperatively.

**Methods:**

A single center cardiac surgery database was queried for patients (aged >/ = 18 years) requiring mitral valve surgery with concomitant CABG or PCI between January 2010 and June 2018. Patients were separated into two groups: mitral valve repair using ring annuloplasty (MVr) and mitral valve replacement (MVR).

**Results:**

A total of 111 patients (median age 66 years, 76% male) underwent an operation for ischemic mitral regurgitation during the study period. (44%) had MVr and 62 (56%) had MVR. Both groups had > 80% concomitant CABG. The MVr group had lower EF (40% vs. 55%, p < 0.01), shorter cardiopulmonary bypass time (117 vs. 164 minutes, p < .01) and shorter aortic cross-clamp time (80 vs. 116 minutes, p < .01). The in-hospital mortality (6% vs. 10%, p = 1.00) and 1-year mortality (14% vs. 18%, p = 0.17) were similar between the groups. Pre-operative left ventricular internal diameter at end-diastole was greater in the MVr group (5.6cm vs. 4.6cm, p < .01). At 1-year, more patients in the MVR group had no or trace regurgitation (29% vs. 61%, p = 0.01), however, the number of patients with moderate or greater mitral regurgitation was similar (6% vs. 12%, p = 0.69). At 2-years, the MVr and MVR groups had no difference in moderate or severe mitral regurgitation (7% vs. 13%, p = 0.68).

**Conclusion:**

Our findings demonstrate similar early mortality and mid-term mitral valve performance, suggesting that MV repair could be a good surgical option in patients with ischemic MR requiring surgical revascularization.

## Introduction

Ischemic mitral regurgitation (iMR) results due to remodeling and/or distortion of the left ventricle following ischemic heart disease that can impair leaflet coaptation, papillary muscle function, and overall mitral valve geometry and competence [1, 2]. iMR has been shown to impact and predict long-term mortality, and surgical revascularization alone is often insufficient [2–5]. Current consensus guidelines suggest revascularization and surgical mitral valve intervention at the same time has a benefit, however, the type of mitral valve intervention (repair or replacement) is still a topic of discussion [6–8]. Surgical mitral valve intervention in the setting of ischemic cardiomyopathy is intended to prevent left ventricular (LV) volume overload and worsening LV remodeling and dysfunction [9–11]. Despite this theoretical benefit, surgical correction of moderate to severe mitral regurgitation in the setting of ischemia has not been shown to improve survival in multiple studies [1, 12–15]. When considering surgical intervention for iMR, the CTSNet moderate and severe iMR trials are often referenced, which concluded that patients who underwent mitral valve repair (MVr) had higher rates of postoperative moderate to severe MR when compared to mitral valve replacement (MVR) [5, 16]. This contradicted previous reports of patients having improved survival and fewer complications following MVr [1, 17–20]. In the present study, we looked at our single-center outcomes for patients who underwent mitral valve repair or replacement for ischemic mitral regurgitation.

## Methods

This study was reviewed by the Institutional Review Board (IRB) and was exempt from approval due to its retrospective nature (45 CFR 46.101(b)). Informed consent was waived for this study (45 CFR 46.116 (D)). Clinical trial registration was also not applicable for this study.

### Study population

A retrospective single-center cardiac surgery database was queried for patients (aged >/ = 18 years) who underwent mitral valve surgery with coronary revascularization between January 2010 and June 2018. Patients who underwent either MVr or MVR with concomitant coronary artery bypass graft (CABG), or who underwent mitral valve surgery in the setting of recent percutaneous coronary intervention (PCI) were included when performed for ischemic mitral valve pathology as evidenced on echocardiography. Patients who had only mitral valve repair in the absence of recent PCI or CABG and patients with mixed mitral valvular etiologies (degenerative plus functional component) were excluded. 93 patients were identified as having CABG with concomitant mitral valve surgery for ischemic mitral regurgitation. 18 patients were identified as having PCI within the previous 6 months of mitral valve surgery. These patients otherwise had optimal medical management. Patients were divided into two groups: those who underwent mitral valve repair using an asymmetric rigid complete annuloplasty ring and those who underwent mitral valve replacement (decision for mechanical vs. tissue valve was made after discussion with patient and risk-profile assessment to determine optimal choice on a case-by-case basis). Annuloplasty ring was undersized by one size. In patients who underwent valve replacement, the posterior leaflet of the mitral valve along with the apparatus was preserved in order to protect the left ventricle geometry. The anterior leaflet was preserved as long as there was no compromise to the size of the prosthesis of left ventricular outflow tract. Subvalvular procedures were not performed. Baseline patient characteristics, demographics, and peri-operative, operative, and postoperative outcomes were collected from available variables provided by the Society of Thoracic Surgeons Adult Cardiac Surgery Database. Additional information on patient outcomes and operative information were obtained by hospital medical record review.

### Primary and secondary outcomes

The primary outcomes were the rate and degree of mitral regurgitation at 1-year and 2-years, as well as freedom from reoperation. This was evaluated by medical record review of 1- and 2- year postoperative echocardiography (ECHO) reports obtained across our three accessible hospital systems, as well as record retrieval from patient cardiology offices. Secondary outcomes were in-hospital survival and 1-year survival. Differences in cardiopulmonary bypass (CPB) time, aortic cross-clamp time, and coronary vessels intervened upon were also analyzed.

### Statistical analysis

The data was divided into 2 groups, patients who underwent mitral valve repair and patients who underwent mitral valve replacement. Baseline characteristics and outcomes were evaluated between the two groups using Wilcoxon tests for continuous variables and Chi-square test for categorical variables. The analysis was performed using SAS software 9.4 (SAS Institute Inc.) at 95% confidence interval level. Continuous variables were reported as mean with interquartile ranges and categorical variables were reported as numbers and percentages.

## Results

### Study population

A total of 111 patients underwent mitral valve surgery for ischemic mitral regurgitation by a single surgeon at our center between January 2010 and June 2018. The median age of the cohort was 66 years, and 76% of the cohort was male. Overall, 62 (56%) had a mitral valve replacement and 49 (44%) had a mitral valve repair. The baseline characteristics of both groups were similar (Table 1) with the exception that the mitral repair group had a lower preoperative median left ventricular ejection fraction (EF), (MVr 40% vs. MVR 55%, p < 0.01) (S1 Data). The MVr and MVR groups had similar rates of moderate and severe mitral insufficiency, both groups with predominantly severe MR (69% and 74%). The MVr group notably had a larger preoperative median left ventricular internal diameter at end diastole (LVIDd) (5.6cm vs. 4.6 cm, p < .01).

**Table 1:** Demographic and perioperative data on patient groups. Data reported as median with interquartile range.

	MVr (n = 49)	MVR (n = 62)	p-value
**Age**	66 (60–71)	64 (60–77)	0.06
**Gender Male**	76% (37)	58% (36)	0.07
**BMI**	28 (25–32)	28 (25–32)	0.94
**Previous Cardiac Surgery**	12% (6)	16% (10)	0.05
**Ejection Fraction (%)**	40 (30–50)	55 (40–60)	< .01
**Previous MI**	49% (24)	63% (39)	0.18
**Diabetes**	37% (18)	19% (12)	0.07
**Hypertension**	90% (44)	87% (54)	0.9
**Hyperlipidemia**	75% (37)	72% (45)	0.9
**Creatinine**	1.0 (0.9–1.2)	1.1 (0.9–1.3)	0.72
**Mitral Insufficiency**			
**Moderate**	31% (15)	26% (16)	0.90
**Severe**	69% (34)	74% (46)	
**CPB time**	117 (103–129)	164 (130–205)	< .01
**Cross-clamp time**	80 (72–91)	116 (92–142)	< .01
**Concomitant CABG**	86% (42)	82% (51)	0.79
**Concomitant valve**	4% (2)	10% (6)	0.46
**OM or LCx intervention**	52% (26)	50% (31)	0.74
**RCA or PDA intervention**	62% (31)	62% (38)	0.83
**LVIDd**	5.6 (5.1–6.2)	4.6 (4.2–5.5)	>.01

MVr = mitral valve repair, MVR = mitral valve replacement, BMI = body mass index

MI = myocardial infarction, CPB = cardiopulmonary bypass, CABG = coronary artery bypass grafting, OM = obtuse marginal artery, LCx = left circumflex artery, RCA =. Right coronary artery, PDA = posterior descending artery, LVIDd = left ventricular internal diameter end diastole

### Primary outcomes

Of the 111 patients reviewed, 93 achieved 1-year follow-up (42 (86%) of MVr patients and 51 (82%) of MVR patients). Of these patients, 72 patients (31 (74%) MVr and 41 (80%) MVR patients) had ECHO information available at this visit. At 1-year, more patients in the MVr group had greater than trace mitral regurgitation (71% vs. 39%), with 65% of MVr patients having mild MR. The number of patients with moderate or severe mitral regurgitation was similar between the MVr and MVR groups (6% vs. 12%, p = 0.69). At 2-years, the MVr and MVR groups had no difference in moderate or severe mitral regurgitation (7% vs. 13%, p = 0.68)(see Table 2). One patient in the MVR group required reoperation 5-years postoperatively for recurrent symptomatic severe MR, which was replaced with a mechanical mitral valve along with concomitant CABG to the RCA.

**Table 2:** Perioperative and ECHO-based outcomes of patients who underwent either mitral valve repair or replacement.

	MVr (n = 49)	MVR (n = 62)	p-value
**In-hospital Mortality**	6% (3)	10% (6)	1.00
**1-year mortality**	14% (7)	18% (11)	0.17
**Patients alive at 1-year**	n = 42	n = 51	
**Follow-up ECHO available at 1-year (n = 72)**	74% (n = 31)	80% (n = 41)	
**None**	10% (3)	34% (14)	0.01
**Trace**	19% (6)	27% (11)	
**Mild**	65% (20)	27% (11)	
**Moderate**	6% (2)	7% (3)	
**Severe**	0 (0)	5% (2)	
**Post-op 1Y MR ≥ Moderate**	6% (2)	12% (5)	0.69
**Post-op 2Y MR ≥ Moderate**	7% (2/29)	13% (5/38)	0.40
**Reoperation**	0	1	

MVr = mitral valve repair, MVR = mitral valve replacement, MR = mitral regurgitation

### Secondary outcomes

The operative mortality (6% for MVr vs. 10% for MVR, p = 1.00) and 1-year mortality (14% for MVr vs. 18% for MVR, p = 0.17) for both groups were similar.

### Intraoperative comparison

Greater than 80% of patients in both groups had a concomitant coronary artery bypass grafting procedure at the time of mitral valve surgery. There were equal rates of additional concomitant valvular surgery between the two groups (4% in MVr patients, 10% in MVR patients, p = 0.46). The MVr group had a shorter average cardiopulmonary bypass (CPB) (117 minutes vs. 164 minutes, p < .01) and aortic cross-clamp time (80 minutes vs. 116 minutes, p < .01)(Table 1). Cardiac vessels intervened upon were similar between groups with ~50% in each group having PCI or CABG to the obtuse marginal artery (OM) or left circumflex (LCx), and 62% in each group having intervention to their right coronary artery (RCA) or posterior descending artery (PDA).

## Discussion

In this study, we describe our experience and outcomes using both mitral valve repair and replacement strategies for ischemic mitral regurgitation. Overall, our patient populations in both groups were similar aside from an expected shorter duration of CPB and cross-clamp time in the repair group. The CTSNet severe iMR trial compared these two surgical techniques in patients with severe MR [21]. Patients who underwent MVr for ischemic MR had a higher rate of moderate or severe MR (32.6% vs. 2.3%, p < .001 at 1-year, 58.8% vs. 3.8%, p < .001 at 2-years) when compared to those who underwent MVR. Additionally, there were more heart failure related adverse events and readmissions related to cardiac disease.

The CTSNet moderate iMR trial compared outcomes from MVr with concomitant coronary artery bypass grafting (CABG) to CABG alone. These results suggested an overall similar survival with decreased rates of moderate to severe MR, but with increased early neurological events and supraventricular arrythmias in the CABG + MVr group [22]. Following this and other various studies, AATS guidelines suggest MVR with preservation of the posterior and anterior chords, or MVr with an undersized complete rigid annuloplasty ring are reasonable to consider for severe iMR, and MVr may be considered in the setting of other indicated cardiac surgery (e.g. CABG) in patients with moderate iMR.

Compared to the CTSNet data from moderate and severe ischemic MR trials, our overall incidence of moderate to severe postoperative MR at 1- and 2-years after MVr is relatively low. While the present study included both patients with preoperative moderate and severe mitral regurgitation, the CTSNet severe iMR trial only looked at patients with severe MR. Despite this difference, the rate of postoperative moderate to severe MR in our MVr patient population (6% at 1-year, 7% at 2-years) was low compared to the CTSNet data (32.6% at 1-year, 58.8% at 2-years). This suggests that an appropriately selected mitral valve repair has good hemodynamic durability when performed well by an experienced surgeon. When compared to patients who underwent MVR in the present study, our MVr patients had a lower preoperative EF and larger LVIDd, possibly suggesting more advanced LV remodeling and worsened baseline disease, however, further analysis would be needed to determine this significance. It is unclear whether this difference in preoperative LV dilation impacted our results. A more dilated LV may benefit from undersized annuloplasty band and reduction in annular dimension, leading to preserved mitral valve function rather than an oversized mitral valve replacement. On the contrary, a more dilated LV may suggest more advanced disease and dysfunction, leading to worsened outcomes with repair or replacement given the unclear promise of reverse LV remodeling. Reverse LV remodeling is a theoretical benefit to MVr or MVR in the setting of iMR. In available studies, however, despite improved rates of moderate to severe mitral regurgitation, the improvement in reverse remodeling varies [22, 23].

Five patients who underwent MVR developed clinically significant mitral regurgitation in follow-up. In review of these five patients, 2 of the patients developed severe mitral regurgitation, 1 secondary to infective endocarditis with paravalvular abscess at 3 years after surgery, and 1 who initially was lost to follow-up, however, presented 6 years later and was found to have severe MR in the setting of new onset atrial fibrillation. The 3 additional patients developed moderate mitral regurgitation. 1 patient developed infective endocarditis, 1 developed complete heart block 3.5 years after being lost to follow-up, during which they were found to have moderate MR, and the final patient was additionally lost to follow-up and found to have central moderate MR after eventually following up with his cardiologist 6 years after surgery.

Furthermore, retrospective studies and various meta-analyses have shown a mix of outcomes after MVr when compared with MVR, suggesting that these outcomes are variable [24–27]. Overall, it is still unclear when MVr is a more appropriate option than MVR, however, a well-performed MVr in an appropriately selected patient can have non-inferior outcomes to MVR, and the criteria for choosing one surgical intervention over the other deserve further discussion and prospective studies.

## Limitations

This study contains the limitations inherent in a retrospective review. While follow-up between groups was similar, the lack of follow-up ECHO data in 35% of the study population limits the overall scope of this study. The low number of patients studied similarly limits the ability to broadly draw conclusions, however, it does offer insight into surgeon-specific experience in performing these procedures. The single-center, single-surgeon nature of this study allows for consistency and expertise in technique and decision-making, to yield results that may not be able to be extrapolated to the greater population. Each case was discussed with the multidisciplinary heart team prior to operation, however, the decision to perform MVr vs. MVR was made intraoperatively at the discretion of the experienced surgeon, and not by a multidisciplinary team. While this may limit the standardization of principles described here, it also suggests that surgeon expertise may play a role in performing a quality repair. Additionally, the Carpentier-McCarthy-Adams IMR ETlogix (Edwards LifeSciences, California, USA) annuloplasty ring was used in all patients who underwent MVr. This may limit the overall conclusions one may draw about mitral valve repair with other devices. This study was performed in patients who experienced mitral regurgitation in the setting of ischemic heart disease and cannot be extrapolated to patients with primary mitral regurgitation.

## Conclusion

Patients with moderate to severe ischemic mitral regurgitation may benefit from mitral valve surgery at the time of coronary revascularization. MVr and MVR have similar hemodynamic outcomes at two years in our population with an overall low incidence of clinically significant MR and equivalent survival. Surgeon experience, regional factors, and careful preoperative and intraoperative decision making may impact the success of both methods of mitral valve surgery. Mitral valve repair can be a durable option in appropriately selected patients and should be considered first in patients with ischemic mitral regurgitation.

## Supporting information

S1 Data (XLSX)
